# Measuring facility capability to provide routine and emergency childbirth care to mothers and newborns: An appeal to adjust for delivery caseload of facilities

**DOI:** 10.1371/journal.pone.0186515

**Published:** 2017-10-19

**Authors:** Stephanie M. Allen, Charles Opondo, Oona M. R. Campbell

**Affiliations:** 1 Department of Population Health, Faculty of Epidemiology and Population Health, London School of Hygiene & Tropical Medicine, London, United Kingdom; 2 Department of Medical Statistics, Faculty of Epidemiology and Population Health, London School of Hygiene & Tropical Medicine, London, United Kingdom; 3 National Perinatal Epidemiology Unit, Nuffield Department of Population Health, University of Oxford, Oxford, United Kingdom; 4 Department of Infectious Disease Epidemiology, Faculty of Epidemiology and Population Health, London School of Hygiene & Tropical Medicine, London, United Kingdom; University of Washington, UNITED STATES

## Abstract

**Background:**

Measurement of Emergency Obstetric Care capability is common, and measurement of newborn and overall routine childbirth care has begun in recent years. These assessments of facility capabilities can be used to identify geographic inequalities in access to functional health services and to monitor improvements over time. This paper develops an approach for monitoring the childbirth environment that accounts for the delivery caseload of the facility.

**Methods:**

We used data from the Kenya Service Provision Assessment to examine facility capability to provide quality childbirth care, including infrastructure, routine maternal and newborn care, and emergency obstetric and newborn care. A facility was considered capable of providing a function if necessary tracer items were present and, for emergency functions, if the function had been performed in the previous three months. We weighted facility capability by delivery caseload, and compared results with those generated using traditional “survey weights”.

**Results:**

Of the 403 facilities providing childbirth care, the proportion meeting criteria for capability were: 13% for general infrastructure, 6% for basic emergency obstetric care, 3% for basic emergency newborn care, 13% and 11% for routine maternal and newborn care, respectively. When the new caseload weights accounting for delivery volume were applied, capability improved and the proportions of deliveries occurring in a facility meeting capability criteria were: 51% for general infrastructure, 46% for basic emergency obstetric care, 12% for basic emergency newborn care, 36% and 18% for routine maternal and newborn care, respectively. This is because most of the caseload was in hospitals, which generally had better capability. Despite these findings, fewer than 2% of deliveries occurred in a facility capable of providing all functions.

**Conclusion:**

Reporting on the percentage of facilities capable of providing certain functions misrepresents the capacity to provide care at the national level. Delivery caseload weights allow adjustment for patient volume, and shift the denominator of measurement from facilities to individual deliveries, leading to a better representation of the context in which facility births take place. These methods could lead to more standardized national datasets, enhancing their ability to inform policy at a national and international level.

## Introduction

Labor, delivery and the first 24 hours after birth are high-risk periods for mothers and babies. It has been argued that reducing mortality among mothers and babies can be achieved only by improving the quality of care, in addition to ensuring coverage and that this feat will require continuous monitoring and assessment—actively using data to inform and guide decisions and actions [1]. While it would be ideal to have data on individual women’s receipt of specific preventive or treatment interventions (the content of care), such data are difficult to obtain where health records are poor. Similarly, health outcomes such as maternal and neonatal mortality are also difficult and expensive to measure in the absence of reliable vital registration; thus, these metrics are frequently not available for monitoring short-term progress [2].

**Fig 1:**
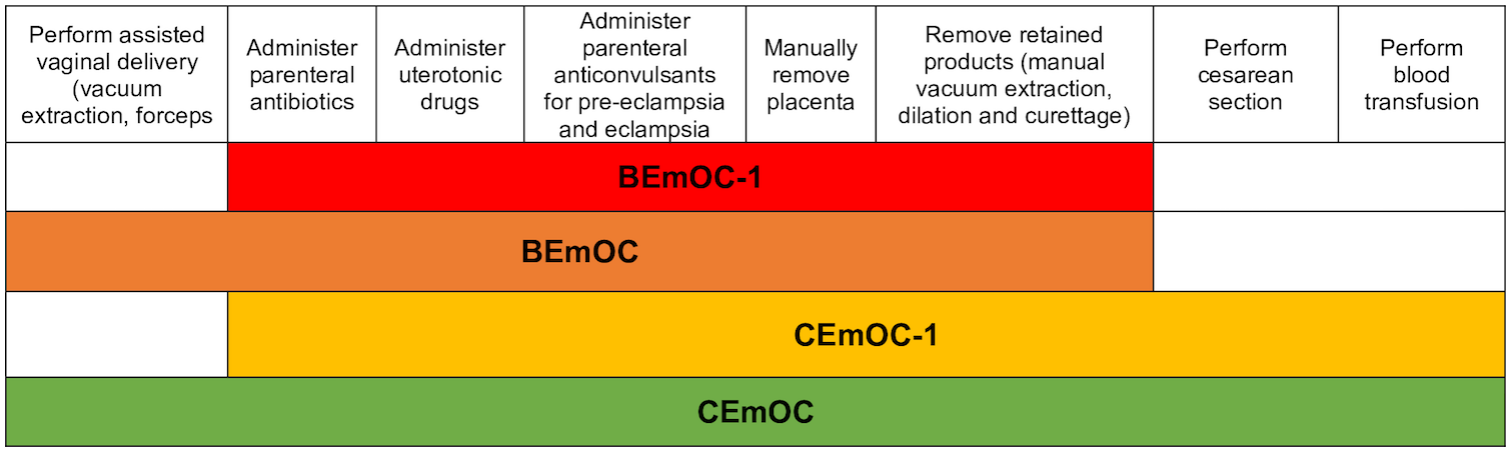
Signal functions and classifications used to identify basic and comprehensive emergency obstetric care. BEmOC includes assisted vaginal delivery, administration of parenteral antibiotics, administration of uterotonic drugs, administration of parenteral anticonvulsants, manual removal of placenta and removal of retained products. BEmOC-1 includes all BEmOC functions except assisted vaginal delivery. CEmOC includes all BEmOC functions in addition to cesarean and blood transfusion capabilities. CEmOC-1 includes all CEmOC functions except assisted vaginal delivery [5–6].

Instead, monitoring childbirth process indicators has been proposed as an alternative, since information about process indicators can guide policies and programs that can subsequently decrease maternal mortality [3]. In 1986, the World Health Organization (WHO)’s *Essential Obstetric Functions at First Referral Level* defined the “essential elements of obstetric care” at the health center, sub-district and district hospital level [4]. While this publication mostly focused on treatment for obstetric complications, it also included an obstetric monitoring function (partograph) and an emergency newborn care function (neonatal resuscitation). In 1997, *Guidelines for monitoring the availability and use of obstetric services* were published by United Nations International Children’s Emergency Fund (UNICEF), WHO and United Nations Population Fund (UNFPA). These *Guidelines* focused on a short list of Emergency Obstetric Care (EmOC) “signal functions”, which are key medical interventions needed to treat obstetric complications that are the leading causes of maternal death worldwide, namely hemorrhage, hypertensive diseases of pregnancy, infection, obstructed labor, and unsafe abortion [3]. While these signal functions did not include every service that should be provided to care for pregnant women, they were intended to “signal” the level of care provided at individual facilities. The EmOC signal functions were further divided into basic (BEmOC) and comprehensive services (CEmOC) [5]. Later, modifications to EmOC criteria were recognized because many facilities did not meet criteria for basic or even comprehensive emergency obstetric care simply because they lacked the ability to perform assisted vaginal delivery with forceps or vacuum, because these skills were not routinely being taught to trainees and therefore not performed [6]. Such facilities were subsequently labeled “BEmOC-1” or “CEmOC-1” indicating that, for example, a given facility meets all BEmOC criteria save for assisted vaginal delivery. The four iterations of EmOC categorization are shown in Fig 1 and an index of all abbreviations utilized in this paper can be found in Table 1.

**Table 1:** Abbreviation index.

BEmNC*	**Basic Emergency Newborn Care**
BEmOC-1*	Basic Emergency Obstetric Care minus one function
BEmOC*	Basic Emergency Obstetric Care
CEmNC*	Comprehensive Emergency Newborn Care
CEmOC-1*	Comprehensive Emergency Obstetric Care minus one function
CEmOC*	Comprehensive Emergency Obstetric Care
**D&C**	Dilation and curettage
**DHS**	Demographic and Health Surveys
EmNC*	Emergency Newborn Care
EmOC*	Emergency Obstetric Care
**HIV/AIDS**	Human Immunodeficiency Virus/Acquired Immunodeficiency Syndrome
**IQR**	Interquartile range
**KMC**	Kangaroo mother care
**NGO**	Non-governmental organization
**NS**	Normal saline
**PPROM**	Premature preterm rupture of membranes
**SARA**	Service Availability and Readiness Assessment
**SPA**	Service Provision Assessment
**UNFPA**	United Nations Population Fund
**UNICEF**	United Nations International Children’s Emergency Fund
**WHO**	World Health Organization

*Further elaboration on which functions are included in this metric can be found in Fig 1.

The EmOC signal functions are captured via health facility assessments such as the Demographic and Health Survey (DHS) Service Provision Assessments (SPA) and the World Health Organization Service Availability Readiness Assessments (SARA).

The great emphasis on management of obstetric emergencies within maternal health metrics over the past three decades has led to a relative neglect in measuring newborn care functions and aspects of routine and preventive care, despite the potential to prevent obstetric complications by focusing on quality routine care [7–8]. In 2012, Gabrysch and colleagues [7] proposed adding new signal functions to facility assessments to expand measurement of emergency neonatal care functions (EmNC) beyond the existing function of neonatal resuscitation, measure provision of routine childbirth care, and assess general facility infrastructure. Nesbitt and colleagues [8] were the first to apply the framework suggested by Gabrysch and colleagues [7]; additionally, for more robust measurement, they suggested measurable tracer items for each signal function, which are the drugs and equipment needed to perform a given signal function.

Facility assessment surveys such as the SPA or SARA sample a smaller fraction of lower level facilities (such as health centers or dispensaries) compared to larger higher-level facilities such as provincial or national hospitals, where they might even include all eligible facilities in a surveyed country. They then employ traditional survey weighting techniques to account for stratification (typically by province and facility type) and cluster sampling. While this method is valuable in ensuring that the facilities included in the study sample are representative of facilities nationwide, its weakness lies in its treatment of individual facilities as the outcome of interest, rather than the means by which care is provided to individual patients. In the well-studied Donabedian Model that enables evaluation of quality in health care, this would be an example of focusing on measurement of a “structure” instead of looking toward an “outcome”. In the Donabedian Model, information from which inferences about quality of care can be classified into three domains: “structure”, “process”, and “outcome” [9]. The Donabedian Model approach is only possible because improved structure leads to increased likelihood of improved process and improved process increases the likelihood of improved outcomes downstream [10]. Thus, these relationships must be established before indicators are used to measure quality of care. In the case of measuring capabilities of childbirth care environments, this means that solely relying on more “upstream” indicators such as facilities’ ability to provide routine or emergency functions could cause some facilities to meet criteria but, in reality, not be able to provide this perceived quality care to patients due to factors unmeasured by these metrics. One crucial dimension that remains uncaptured by current metrics is the delivery caseload (or number of deliveries in a given period of time) in each facility.

In recent decades, national preparedness to provide emergency obstetric care has been measured using EmOC facility density, with geographical areas meeting the benchmark if at least five EmOC facilities were present for every 20,000 births in the area [5]. This specific indicator is problematic for the same reason that traditional survey weighting techniques can be problematic: it ignores the crucial dimensions of facility size and delivery caseload. Facility size and number of deliveries taking place in a given facility were identified as important factors in a paper examining the correlation of traditional health-system output indicators (such as density of facilities able to provide EmOC) with system impact measurements, such as maternal mortality [11]. While Zambia and Sri Lanka performed similarly in terms of EmOC facility density (thus “meeting criteria” as mentioned previously), maternal mortality rates drastically differed, illustrating a poor correlation between the two measurements: EmOC facility density and one significant outcome that EmOC facility density attempts to predict, maternal mortality. Thus, the indicator of EmOC facility density had “low discriminatory power”, as it failed to differentiate between a low-maternal-mortality country and one with a higher maternal mortality rate. However, the authors point out that interpreting these results in the absence of knowing the size (or, presumably, of delivery caseload) could be the reason why the indicator did not perform well, “as it treats large hospitals with thousands of deliveries per year the same as facilities with a few beds”. Furthermore, the authors note that the facilities in Sri Lanka were much larger than those in Zambia, possibly helping to explain the seemingly different depictions of delivery preparedness produced by two countries meeting the same benchmarks.

Without adjusting for the numbers of deliveries within each facility, assessing the percentage of facilities capable of performing given functions such as EmOC gives a picture of delivery preparedness that does not correctly depict the childbirth care environment for mothers and newborns using facilities. In order to transition from facility-centered to birth-centered monitoring and evaluation, we must develop and utilize metrics that enable facility assessment data to be adjusted for delivery caseload. This paper utilizes the framework of Gabrysch and colleagues [7] to develop this new approach to summarizing and monitoring the facility childbirth environment at a global level. To achieve this, we (i) examined facilities’ ability to provide routine and emergency childbirth care for mothers and newborns, (ii) examined the distribution of deliveries by facility level and care capability, and (iii) assessed the usefulness of a weighting method that would allow data to be adjusted for delivery caseload, giving more statistical weight to facilities performing more deliveries and less statistical weight to those performing fewer deliveries.

We used data from Kenya to illustrate our approach. According the 2008–09 Kenya DHS, maternal mortality ratio in Kenya for the period 1998–2009 was 488 maternal deaths per 100,000 live births and neonatal mortality rate of 31 deaths per 1000 live births from 2008–09 [12]. In the five years preceding the 2008–09 Kenya DHS, 43% of births took place in a health facility [13].

## Methods

The SPA surveys are national-level assessments of health system assets that “collect information on the overall ability of facility-based health services in a country and their readiness to provide those services” [14]. Data collection tools utilized for the SPA survey include facility audit questionnaires, exit interviews with clients, health worker/provider interviews and observations of specific types of health visits, such as antenatal care, family planning or sick child. Our analysis only included data from the facility audit’s inventory questionnaire, which was designed to measure readiness indicators and several other developed indicators in maternal and child health. These health service readiness indicators are a set of tracer indicators that help in “measuring and tracking progress in health system strengthening” [14]. The Kenya 2010 SPA included annual numbers of births taking place in each facility included in the survey, although it should be noted that data for this variable were not collected in SPA surveys of other countries at the time. The SPA protocol required interviewers to interview the most knowledgeable person in the facility for each particular service or system component being evaluated, defined as “manager, person in-charge of the facility or most senior health worker responsible for client services”.

The Kenya 2010 SPA was comprised of a sample of public, private, NGO and faith-based facilities. The Kenya Essential Package for Health indicates six levels of healthcare delivery: tertiary/referral hospitals (level 6), provincial hospitals (level 5), district hospitals (level 4), health centers, maternities (level 3), dispensaries, clinics (level 2) and the community [12]. The sampling frame was a Master Facility List with 6,192 functioning health facilities, including all hospital types (tertiary/referral, provincial, district, sub-district, “other”), health centers, maternities, dispensaries, clinics and voluntary counselling and testing centers. A complex survey-sampling strategy was used that required sample weights to be applied for the sample to be nationally representative of all health facilities in Kenya. Of the 703 facilities sampled, 695 (99%) participated in the assessment. Hospitals, health centers, maternities and stand-alone voluntary counselling and testing centers were over-sampled, as they are smaller in number nationwide and provide most of the maternal health and HIV/AIDS care, which were objectives of measurement in the survey. Data were weighted to ensure that the contribution of each facility to the sample reflected the relative proportions of all facility types in Kenya. Further details of the sampling and data collection are described in the Kenya SPA report [12]. Overall, SPA data quality was very good, and few data were missing on signal function provision. Only 403 facilities provided childbirth care (58%) and these were the facilities ultimately included in our analysis. Data on delivery caseload (number of deliveries occurring in the facility in the twelve months prior to survey) were missing in 3% of facilities; these facilities were excluded in the delivery caseload weighted analyses because the delivery caseload weight variable could not be computed.

### Quantifying routine childbirth and emergency obstetric and newborn functions

We measured EmOC provision using previously developed criteria in which a facility was deemed capable of performing a signal function if it had been performed in the facility within the three months prior to survey [15–16]. BEmOC-1 and CEmOC-1 categories were created in an effort to not recognize facilities that met all criteria except assisted vaginal delivery, as providers in many countries are not trained in how to provide this function [6,15–16].

There is little experience to date examining the routine functions proposed by Gabrysch and colleagues [7], and SPA surveys have not explicitly set out to collect data on provision of most of the routine functions. For this reason, the criteria used in this study relied largely on the presence of tracer items suggested by Nesbitt and colleagues [8]. Some proposed functions were not captured in our analysis because the SPA did not include any related tracer items, such as: “alternative feeding if baby is unable to breastfeed”, “application of [baby] eye ointment”, “delivery companion allowed”, “weigh baby” and “safe administration of oxygen to newborns”. While the measurement of most signal functions is self-evident from the description in the tables, some classifications varied by level of facility, namely referral and water requirements. Specifically, facilities that met CEmOC criteria were not required to have referral capability because they were considered to offer the highest level of care and were not expected to refer. Similarly, as proposed by Benova and colleagues [17], hospitals were required to have piped water in the childbirth service area to meet the clean water requirement, whereas non-hospitals were only required to have piped water in some part of the facility, as we judged water could be quickly retrieved from other areas of a small facility when needed.

National Referral Hospitals (n = 2) were combined with Provincial Hospitals (n = 7) due to small sample size, and clinics (n = 103) were combined with dispensaries (n = 147) due to their theoretically similar level of care within the Kenya Essential Package for Health framework [12].

### Analysis

As described previously, the survey sampling was complex, and data needed to be weighted for analysis to achieve national and regional representativeness. We did this using the *svyset* command in Stata 13/SE (StataCorp, TX, USA) and termed these analyses as having used “facility weights”. This is the approach used in SPA reports and previous literature.

**Fig 2:**
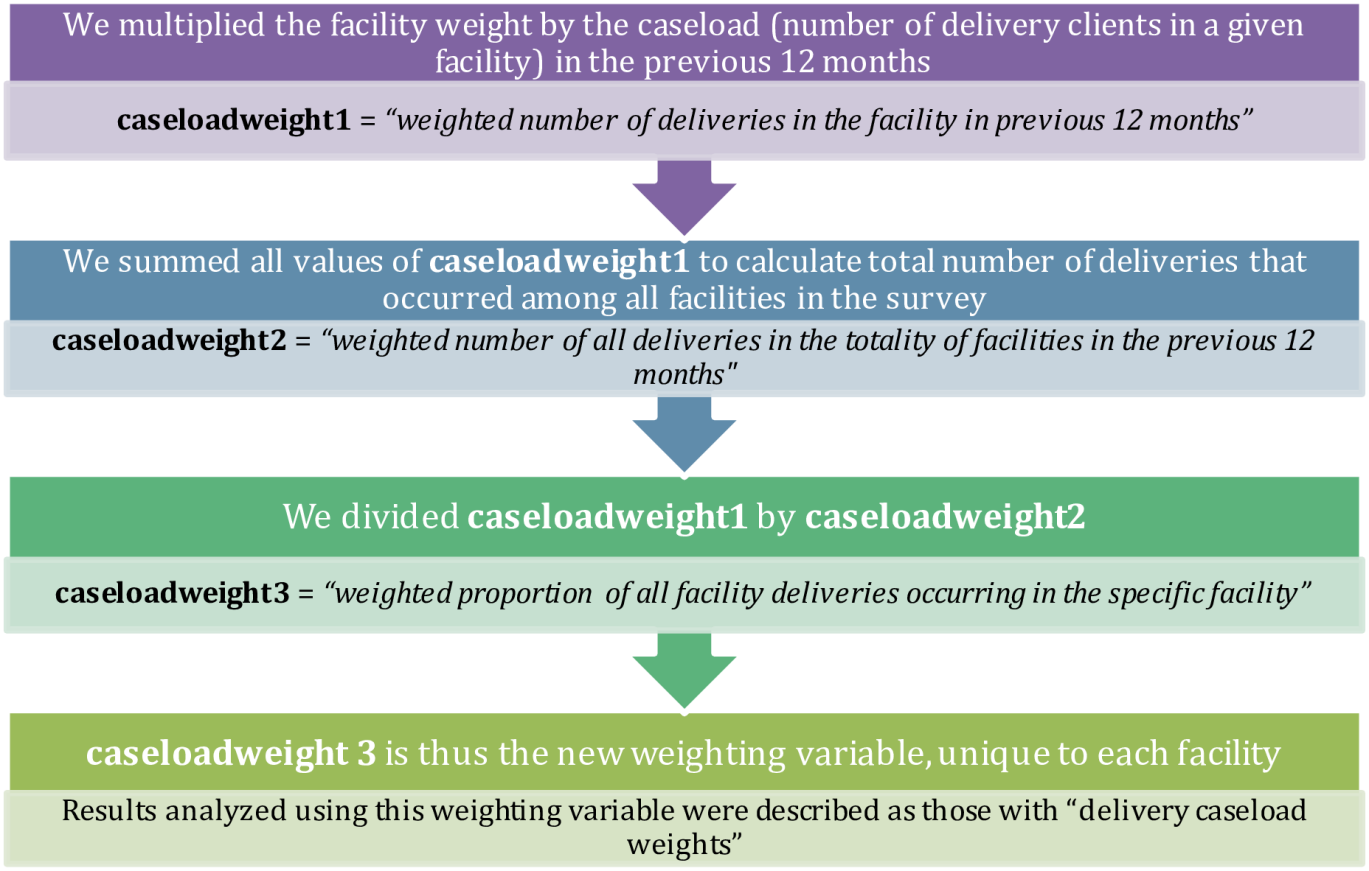
Delivery caseload weight calculation procedure for each facility. Each step in the chart signifies a separate mathematical step, ultimately showing how one can incorporate delivery caseload into survey weight values. A worked example is found in S1 Table.

We further analyzed the data in terms of the delivery caseload in individual facilities. The number of deliveries varied greatly both within and between facility types, and we were interested in describing not just what facilities could do, but what the environment was like for most facility births. For this aspect of the analysis, we created a weighting variable that accounted for both the facility weight and the delivery caseload (measured by the number of deliveries that occurred in the facility in the previous twelve months). As each facility included in this part of the analysis had a unique annual delivery caseload value, each facility subsequently had a unique “delivery caseload weight” value. We created these unique delivery caseload weight values using the following procedure found in Fig 2. A worked example and interpretation of this procedure for one facility can be found in S1 Table.

Measure DHS granted permission to use the dataset; the London School of Hygiene and Tropical Medicine gave ethical approval for secondary data analysis.

## Results

**Fig 3:**
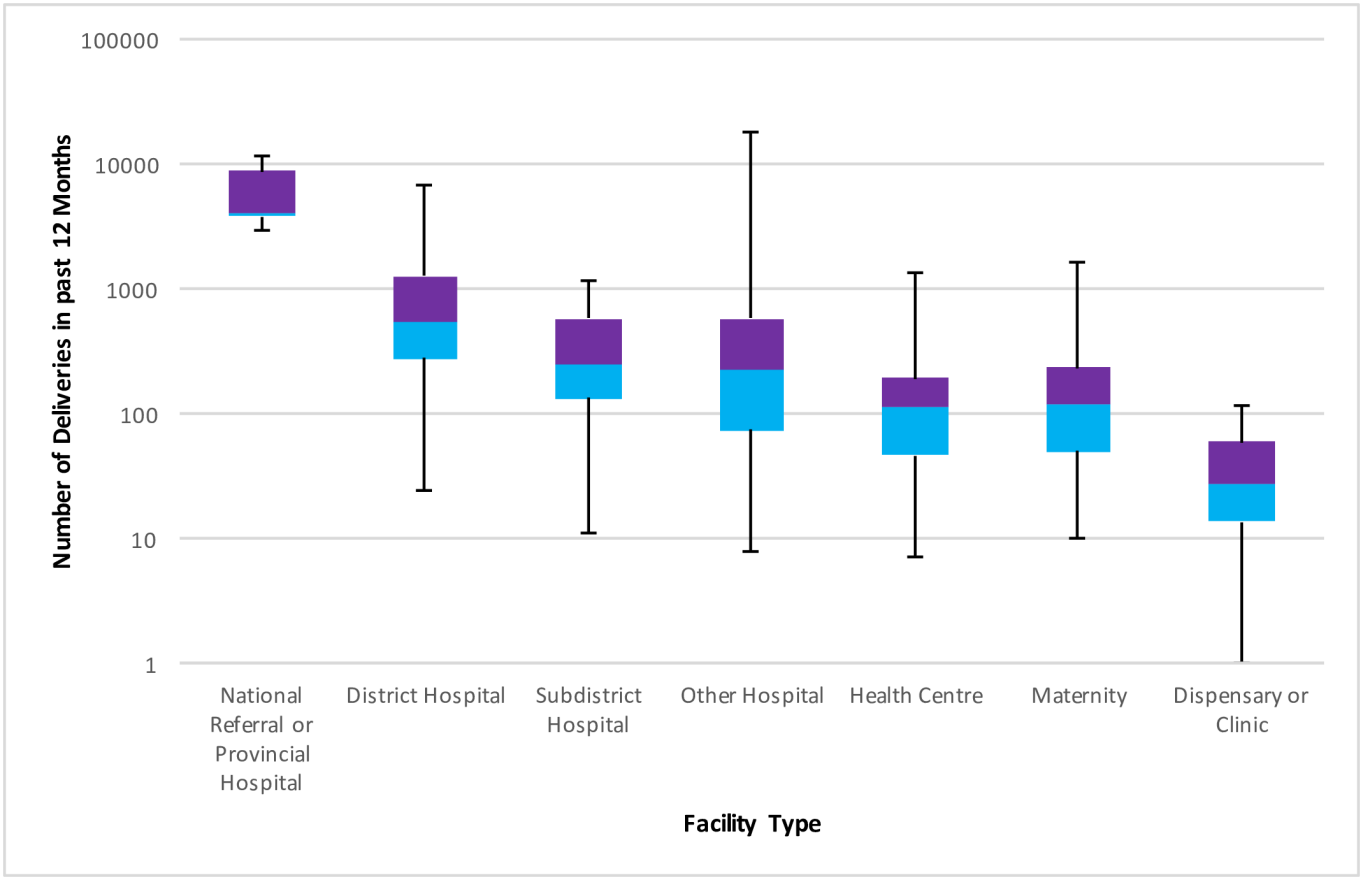
Boxplot showing distribution of number of deliveries across facility type. Median, interquartile range, minimum and maximum number of deliveries in each facility type are displayed.

The numbers and distribution of facilities (facility weights) providing childbirth care is described in Table 2, as are the median, interquartile range (IQRs) and minimum and maximum number of deliveries taking place in each facility type in the previous twelve months. These data are also illustrated in Fig 3. Table 2 and Fig 3 both show that facilities higher up the referral chain tended to have more deliveries.

**Table 2:** Distribution of deliveries in the 2010 Kenya SPA by facility level.

Facility Level	Proportion of facilities (%)*	Number providing childbirth care (% of total number of facilities in that category)	Median number of deliveries in previous 12 months (IQR)^#^	Range in number of deliveries in past 12 months
National Referral or Provincial Hospital	0.3	9 (100%)	4154 (3857–8712)	3021–11531
District Hospital	1.9	71 (100%)	558 (280–1279)	24–6936
Sub-district Hospital	2.0	63 (100%)	245 (134–575)	11–1174
“Other” Hospital	3.1	96 (90%)	227 (75–580)	8–17899
Health Center	11.5	79 (83%)	114 (47–194)	7–1322
Maternity	2.4	44 (85%)	120 (50–236)	10–1650
Dispensary or Clinic	78.1	41 (13%)	27 (14–60)	1–117

* This column does not add up to 100%, as we excluded Voluntary Counselling and Testing Centers (0.7% of sample) from the analysis, as none offered childbirth care.

^#^Data were missing from a total of 11 (3% weighted) facilities about the delivery caseload in the previous 12 months.

### Emergency childbirth care functions

Table 3 details the availability of EmOC and EmNC across facility type, including nested percentages for tracer items needed to perform the function. More facilities were capable of providing parenteral oxytoxics (65%) than any other EmOC function; assisted vaginal delivery capability was the least common (3%). More facilities were capable of providing intravenous (IV) fluids to the newborn (87%) than any other EmNC function. Facilities were least equipped to provide corticosteroids to women in preterm labor (21%).

**Table 3:** Emergency childbirth care capability by facility level.

Signal Function	Criteria (performance or presence of observed tracer items)	Facility performance by level of facility (%)							
		National/Provincial Hospital	District Hospital	Sub-district Hospital	“Other” Hospital	Health Center	Maternity	Clinic or Dispensary	Total Performance
Basic EmOC#									
Parenteral antibiotics	Performed in past 3 months (+ Injectable ampicillin/amoxicillin or gentamicin)	100 (50)	88 (48)	76 (38)	84 (44)	51 (19)	67 (51)	31(11)	52 (24)
Parenteral oxytocin	Performed in past 3 months (+ Injectable oxytocin)	100 (100)	92 (80)	90 (61)	96 (84)	81 (65)	85 (78)	75 (54)	82 (65)
Parenteral anticonvulsants	Performed in past 3 months (+ Injectable diazepam or magnesium sulphate)	100 (87)	64 (63)	22 (20)	57 (50)	14 (11)	24 (22)	5 (5)	20 (18)
Manual removal of placenta	Performed in past 3 months	90	72	52	55	38	33	12	33
Removal of retained products	Facility is “able to perform function” (+ Functioning vacuum aspirator or D&C kit)	100 (100)	89 (71)	88 (58)	93 (81)	55 (40)	72 (59)	30 (15)	55 (40)
Assisted vaginal delivery	Performed in past 3 months (+ Functioning ventouse vacuum extractor)	59 (51)	6 (6)	3 (3)	21 (20)	0 (0)	2 (2)	0 (0)	3 (3)
Comprehensive EmOC									
Blood transfusion	Performed in past 3 months	100	58	8	49	4	23	0	13
Caesarean section	Performed in past 3 months	88	52	7	69	1	31	0	14
Basic EmNC									
Antibiotics to mother if premature preterm rupture of membranes (PPROM)	Injectable ampicillin/amoxicillin or benzylpenicillin	60	63	54	44	46	80	42	48
Corticosteroids in preterm labor	Injectable dexamethasone in the pharmacy	54	31	14	84	13	55	5	21
Resuscitation with bag and mask	Facility has performed function in past 3 months (+bag or tube + mask for baby)	100 (90)	85 (81)	64 (59)	66 (60)	44 (39)	57 (46)	18 (11)	42 (35)
Kangaroo Mother Care (KMC) for premature or small babies	Practice is routine for all babies##	94	62	49	48	57	49	41	50
Injectable antibiotics	Injectable ampicillin/amoxicillin or gentamicin	50	55	52	50	25	40	73	44
Comprehensive EmNC									
Intravenous fluids	Intravenous infusion set & IV solutions (NS) in childbirth service area	100	91	85	96	90	88	81	87

The percentage of facilities of a given type that performed a given function in the previous three months is represented by the first number; the percentage of facilities that performed the function in the previous three months AND had the tracer items necessary to perform the function at the time of survey are represented by the nested percentage in parentheses. We term this the percentage of facilities capable of performing a function.

^#^ All tracer items in this category must be present in the room where deliveries take place or in an adjacent room

^##^ The Kenya SPA did not specifically measure KMC, but did measure “as soon as possible after birth the baby is put in skin contact with the mother” and was asked for all babies, not specific to preterm/very small babies.

### Routine childbirth care functions

Table 4 details the availability of routine care capability by facility level, including nested percentages for tracer items needed to perform the function. Capacity to perform a function generally decreased as the level of facility decreased. Most facilities (83%) had 24-hour childbirth service availability. Nearly all facilities had adequate communication tools and latrines or toilets available for patients. About three-quarters had electricity. While all facilities providing childbirth services had a source of water, only 46% had running water in the childbirth service area. Only 15% of facilities expected to need to refer if necessary had blank referral forms and an ambulance. About half of facilities displayed the tracer items necessary for monitoring labor and infection prevention during labor. The three phases of active management of third stage of labor were reportedly performed routinely by between 45% and 81% of facilities. Regarding routine newborn care, facilities were poorly equipped to provide thermal protection, but performed well in other categories. While drying the baby after birth and keeping the baby warm were routine in 98% of facilities, towels and blankets were present in the childbirth service area of less than one-third of facilities.

**Table 4:** Routine childbirth care capability by facility level.

Signal function	Observed tracer items (corresponding protocols/drugs/equipment)	Facility performance by level of facility (%)							
		National/ Provincial Hospital	District Hospital	Sub-district Hospital	“Other” Hospital	Health Center	Maternity Home	Clinic or Dispensary	Total Performance
General Requirements									
Service availability 24/7		100	100	100	100	87	97	66	83
Infrastructure									
Communication tools	Radio or telephone	100	96	94	97	88	96	93	92
High quality referral system	Referral form (+ Ambulance)	n/a	53 (40)	56 (44)	64 (43)	45 (16)	43 (24)	15 (0)	35 (15)
Electricity, any type		100	99	94	100	92	96	63	83
Toilet or latrine	Functioning latrine for clients	100	100	99	100	96	100	100	99
Water supply	Piped or running water	100	92	95	92	44	52	17	46
Routine Childbirth Care (Maternal)#									
Monitoring and management of labor using partograph	Blank partograph + fetoscope (pinard or electric)	100	85	84	79	72	61	39	61
Infection prevention measures during childbirth (hand-washing, gloves)	Clean water source + hand soap + gloves (latex or non- latex)	81	57	61	72	30	31	9	30
Active management of 3^rd^ stage of labor									
Routine injection of oxytocin within one minute of delivery	Practice is routine** (+ Injectable oxytocin/syntocin or ergometrine/methergine with valid date)	84 (84)	75 (64)	60 (44)	58 (52)	65 (53)	58 (51)	38 (30)	54 (45)
Controlled cord traction	Practice is routine** (+ Cord clamp/ties and scissors/blade)	100 (100)	92 (86)	91 (82)	95 (94)	85 (79)	75 (71)	83 (66)	86 (76)
Uterine massage after delivery of placenta	Practice is routine**	100	92	88	87	86	88	70	81
Routine Childbirth Care (Newborn)#									
Thermal protection									
Drying baby immediately after birth	Practice is routine** (+ Towel for baby)	100 (86)	98 (24)	99 (29)	100 (65)	99 (25)	98 (58)	97 (20)	98 (30)
Skin-to-skin with mother	Practice is routine**	94	62	49	48	57	49	41	50
Wrapping	Practice is routine** (+ Blanket for baby)	100 (35)	98 (11)	99 (15)	100 (54)	99 (21)	98 (54)	97 (24)	98 (27)
No bath in first 6 hours	Practice is routine**	100	97	96	82	95	82	94	93
Immediate and exclusive breastfeeding	Practice is routine**	92	97	99	92	97	93	93	95
Hygienic cord care (cutting with sterile blade)	Scissors/blade	100	93	92	100	92	93	94	94

^#^All tracer items in this category must be observed in the room where deliveries take place or in an adjacent room.

** Practices were considered to be routine if the facility representative interviewed endorsed the practice as routine.

The percentage of facilities of a given type that performed a given function in the previous three months is represented by the first number; the percentage of facilities performed a function in the previous three months AND had the tracer items necessary to perform the function at the time of survey are represented by the nested percentage in parentheses; we term this the percentage of facilities capable of performing a function.

### Summarizing facility preparedness across the continuum of care

Table 5 summarizes Tables 3 and 4. Between 11% and 13% of facilities met routine care capabilities in each category. Only 6% of facilities met the BEmOC-1 criteria used in this study and 3% met BEmNC criteria.

**Table 5:** Availability of general requirements and facility capability of routine and emergency childbirth care, by facility level.

Facility Level	General Require- ments %	ROUTINE %		EMERGENCY %				Total meeting all functions at basic level^#^ %
				Obstetric		Newborn		
		Maternal	Newborn	BEmOC (BEmOC-1)	CEmOC (CEmOC-1)	BEmNC	CEmNC	
National or Provincial Hospital	100	65	35	24 (37)	24 (24)	37	37	9 (9)
District Hospital	43	26	8	3 (20)	2 (12)	5	5	0
Sub-district hospital	37	21	11	1 (6)	1(2)	3	3	0
“Other” hospital	42	35	21	6 (19)	3 (13)	10	10	0 (2)
Health Center	5	14	7	0 (3)	0	3	2	0
Maternity	20	13	19	0 (16)	0 (10)	7	7	0
Clinic or Dispensary	0	3	9	0 (1)	0	0	0	0
Total	13	13	11	1 (6)	3	3	3	0.07 (0.23)

^#^ Meaning that the facility met all requirements, including BEmOC, BEmNC, and routine care functions but not necessarily CEmOC or CEmNC.

In parentheses is the percentage of facilities that met all criteria if the assisted vaginal delivery requirement was excluded.

### Examining facilities by delivery caseload

**Fig 4:**
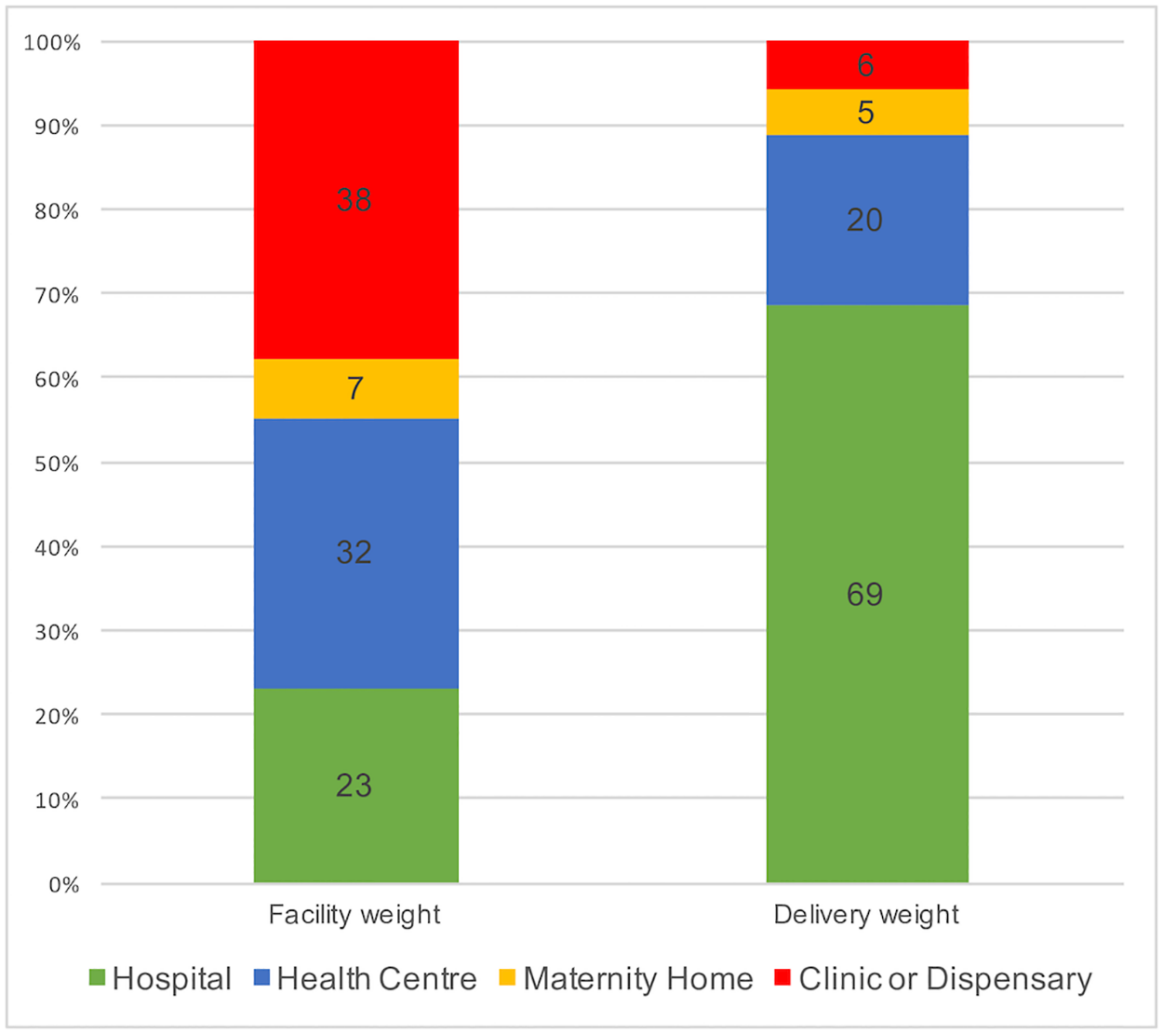
Re-calibrating our measurements: Percentage of facilities in each category vs. percentage of births that occurred in each type of facility (delivery caseload weight).

**Fig 5:**
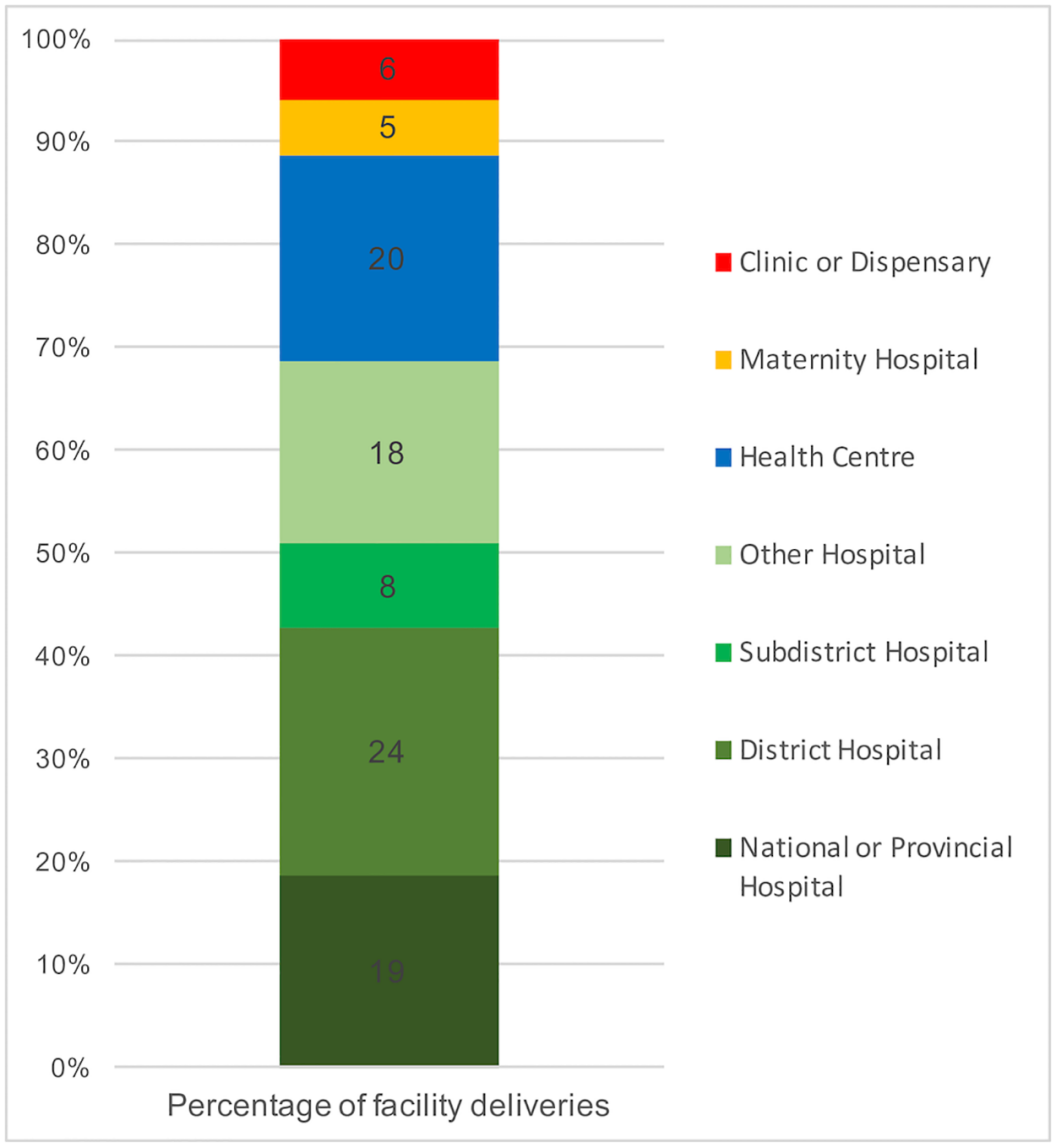
Where are the facility births occurring? Percentage of facility deliveries occurring in each level of facility.

While clinics and dispensaries comprised a sizable proportion of facilities (38%), relatively few deliveries (6%) occurred there. Conversely, hospitals comprised 23% of facilities, but were the location of 69% of the deliveries. Figs 4 and 5 demonstrate the capabilities of facilities in which deliveries took place.

**Fig 6:**
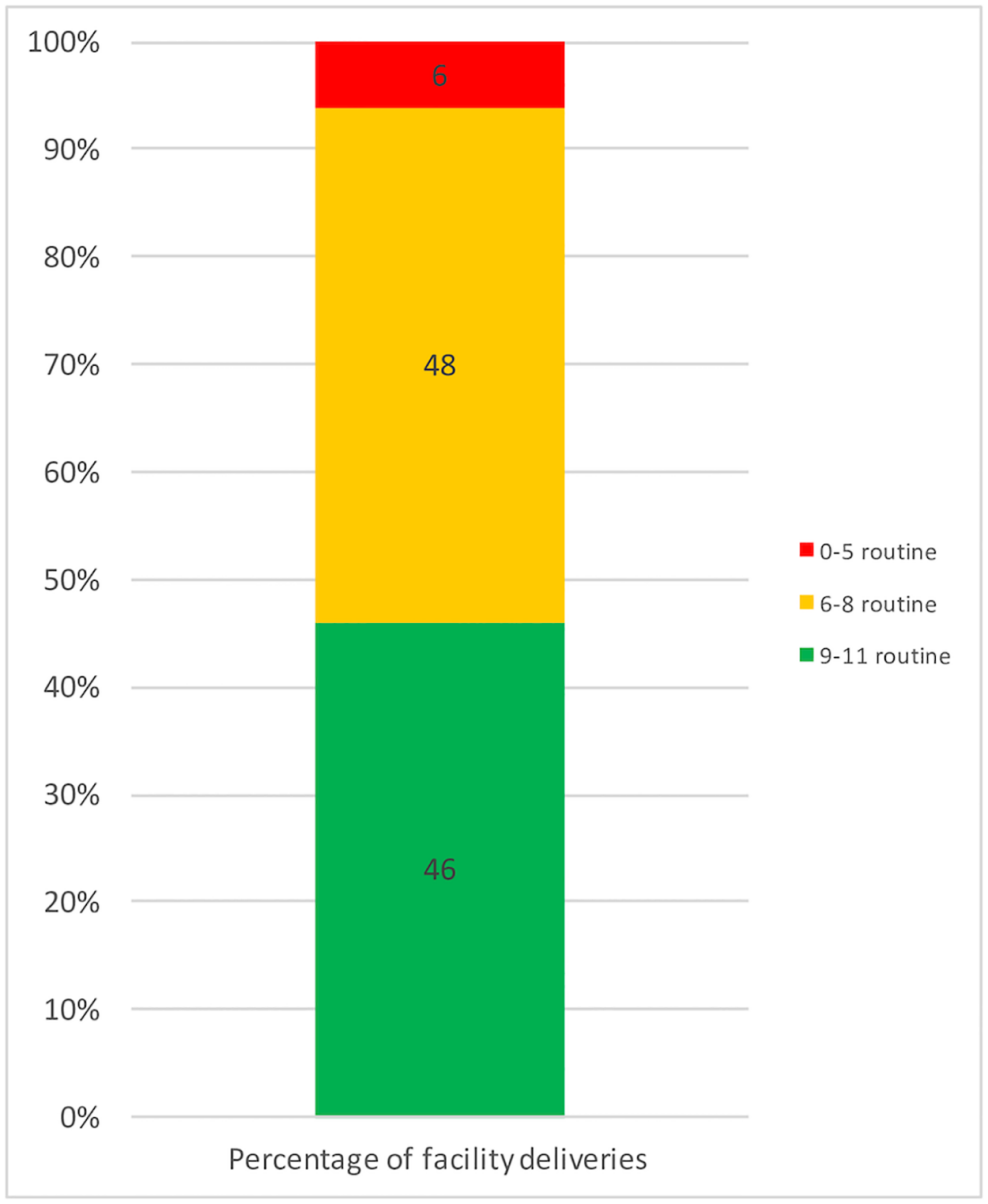
Percentage of facility births taking place in each routine childbirth care capability category.

Roughly half (46%) of facility deliveries occurred in a facility that was equipped to perform at least 9 of the 11 routine childbirth functions for mother and newborn; 6% occurred in a facility that could perform fewer than five routine functions (Fig 6).

### Examining dimensions of quality care using delivery caseload weights

When the delivery caseload weights were applied, the overall picture for facility capability of the settings where births took place improved in every category (Table 6). Among general requirements, greatest improvements were for the referral systems (15% to 43%) and water supply (46% to 81%). Among routine maternal functions, the greatest improvement was seen for infection control (30% to 65%). Among newborn functions, there was little improvement, aside from percentage of facilities capable of drying the baby immediately after delivery (30% to 54%). Among BEmOC functions, improvement was greatest in capability of providing assisted vaginal delivery, followed by parenteral anticonvulsants and least for parenteral oxytocin. Among EmNC functions, greatest improvement was seen in neonatal resuscitation and provision of corticosteroids.

**Table 6:** Difference in facility preparedness, comparing facilities with births in facilities (delivery caseload weight used).

Childbirth Care Function	Proportion of facilities meeting criteria (Facility weight) %	Proportion of total facility births taking place in a facility meeting criteria (Delivery caseload weight) %	Difference in proportion (Deliveries%—Facility%)
General Requirements			
Service availability 24/7	83	98	+15
Infrastructure			
Communication tools	92	96	+4
High quality referral system	15	56	+41
Electricity, any type	83	98	+15
Toilet or latrine	98	98	0
Water supply	46	81	+35
Total	13	51	+38
Routine Childbirth Care (Maternal)#			
Monitoring and management of labor using partograph	61	85	+24
Infection prevention measures during delivery (hand-washing, gloves)	30	64	+34
Active management of 3^rd^ stage of labor			
Routine injection of oxytocin within one minute of delivery	45	63	+18
Controlled cord traction	76	87	+11
Uterine massage after delivery of placenta	81	91	+10
Total	13	36	+23
Routine Childbirth Care (Newborn)#			
Thermal protection			
Drying baby immediately after birth	30	54	+24
Skin-to-skin with mother	50	60	+10
Wrapping	27	30	+3
No bath in first 6 hours	93	94	+1
Immediate and exclusive breastfeeding	95	95	0
Hygienic cord care (cutting with sterile blade)	94	97	+3
Total	11	18	+7
Basic EmOC#			
Parenteral antibiotics	24	46	+22
Parenteral oxytocin	65	87	+22
Parenteral anticonvulsants	18	61	+43
Manual removal of placenta	33	69	+36
Removal of retained products	40	77	+37
Assisted vaginal delivery	3	30	+27
Total BEmOC-1	6	46	+40
Basic EmNC			
Antibiotics to mother if preterm or prolonged PROM	48	52	+4
Corticosteroids in preterm labor	21	46	+25
Resuscitation with bag and mask	35	76	+41
KMC for premature/very small babies	50	59	+9
Injectable antibiotics	44	51	+7
Total	3	12	+9
Comprehensive EmONC			
Blood transfusion	13	59	+46
Caesarean section	14	58	+44
Intravenous fluids	87	90	+3
Total	1	6	+5

^#^All tracer items in this category must be present in the room where deliveries take place or in an adjacent room

**Fig 7:**
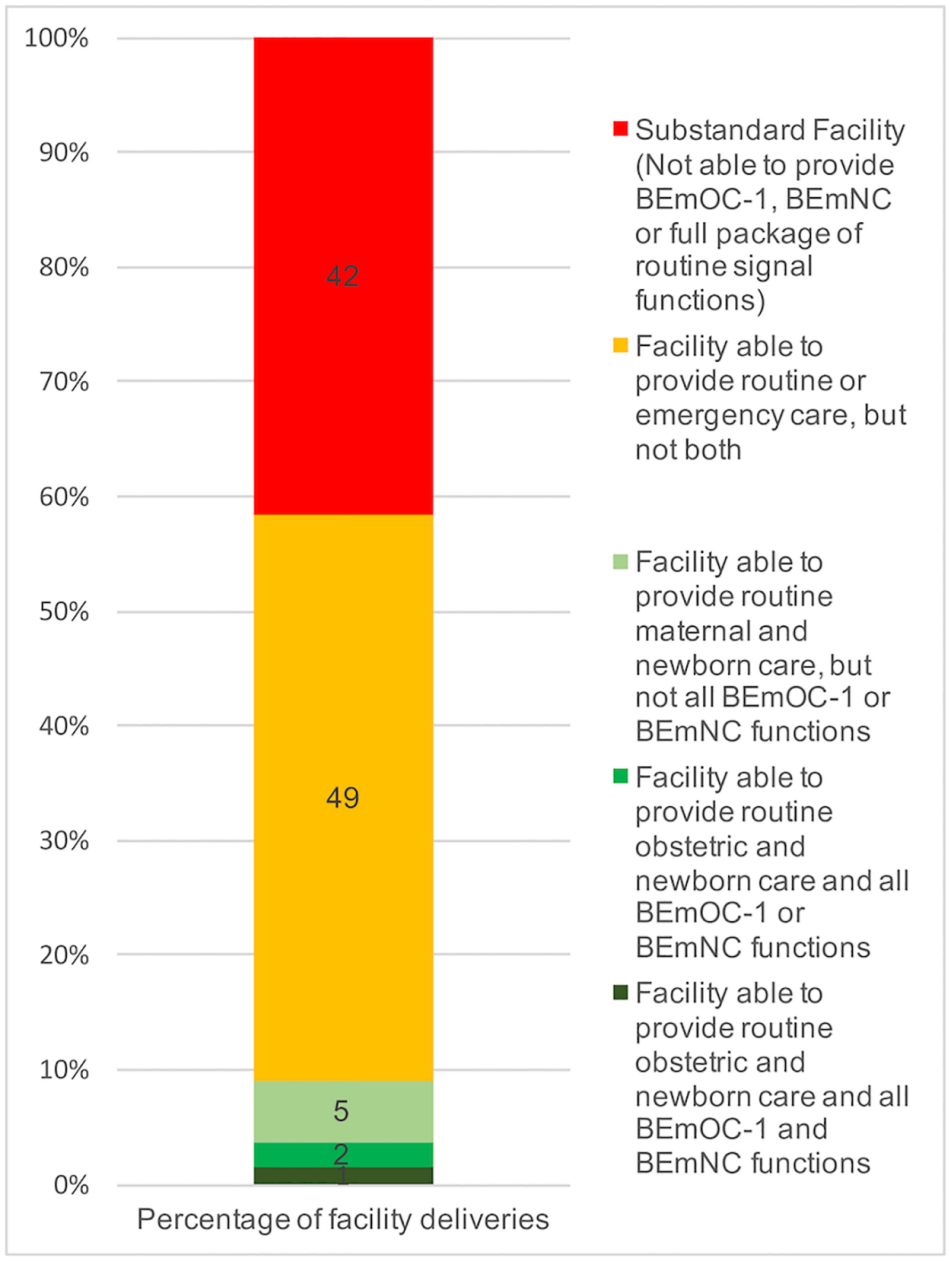
Where the facility births are occurring, by childbirth care capability.

Fig 7 shows the overall routine and emergency capabilities of facilities. Nationally, over 40% of facility births occurred in a facility that was not equipped to provide the full package of routine or emergency childbirth care. Only 1.5% of facility births nationwide took place in a facility equipped to perform all infrastructure, routine, BEmNC, and BEmOC-1 functions.

## Discussion

We found that, nationally, Kenyan facilities met general infrastructure requirements in 13% of facilities, BEmOC-1 capability in 6%, BEmNC in 3%, routine maternal care in 13%, and routine newborn care in 11%. Only 0.23% of facilities met all requirements. However, higher-capability facilities conducted more deliveries on average, and applying delivery caseload weights showed that 51% of births took place in a facility that met all general infrastructure requirements, 46% in a facility meeting BEmOC-1, 12% in a facility meeting BEmNC, 36% in a facility capable of routine maternal care and 18% in a facility capable of routine newborn care. Despite this improvement in the picture of childbirth care, fewer than 2% of births took place in a facility equipped to provide the full spectrum of emergency and routine maternal and newborn care.

To our knowledge, this is the first paper to utilize the Gabrysch framework [7] with routinely collected SPA data to go beyond the EmOC signal functions and explore facility capability and routine signal functions for mothers and newborns. This is essential to monitoring strategies in maternal and newborn health, as proper routine care can prevent complications and thus reduce the need for emergency interventions [7–8]. Evaluating the feasibility of measuring routine care functions in a fashion that is similar to how emergency obstetric functions have been measured for many years was an essential part of our method. We also aimed to demonstrate the usefulness of the delivery caseload weights method for elucidating the picture of delivery preparedness broadly, not just in emergency preparedness.

This is also the first study using a nationally representative dataset to examine the availability of the necessary tracer items for signal functions suggested by Nesbitt and colleagues [8]. We believe that adding tracer items to the criteria excluded facilities that may have been labeled as capable of performing an individual function by virtue of performing it in the previous three months, but would not be prepared to perform the function if a patient had needed the intervention in that moment.

Furthermore, to our knowledge, we are the first to develop the technique adjusting for delivery caseload when looking at childbirth services. Because the unique delivery caseload value from each facility is factored into its individual weighting variable (meaning that each individual facility has a unique weight value in the survey), we believe that this method provides a more accurate representation of childbirth care than when data from individual facilities are aggregated in regional and national surveys stratified only by facility type and province, for example. This would mean that, in the Kenya SPA Survey, that all district hospitals (or any given facility type) in a particular province contribute the same amount of weight to the survey, regardless of facility utilization differences. We believe that factoring in the delivery caseload is an important methodological step that enables investigators to adjust for delivery caseload when assessing delivery preparedness in aggregated national datasets where facility utilization differences can be particularly opaque. Disaggregating datasets can illuminate highly inequitable distributions in facility preparedness [11] and measures of facility utilization, as in our study, and can be particularly useful in identifying gaps in health systems. While this methodology may have less utility in informing resource allocation at a national or subnational level, its merit lies in tracking trends in facility capabilities over time and enabling cross-comparison of multiple countries with differing healthcare system structures. For example, this methodology could better enable cross-national comparisons when a given facility type (e.g., health center) may be expected to handle different delivery caseloads from one country to another. The approach we have developed is increasingly being adopted in multi-national analyses [18–19].

Our study had several important limitations. One of particular concern is the validity of the chosen signal functions themselves. While the framework by Gabrysch and colleagues seemingly measures important dimensions of routine and emergency delivery care, with the exception of the EmOC signal functions and neonatal resuscitation, the signal functions were developed through systematic literature review and soliciting opinion from 39 maternal and newborn health experts and have not been validated [7]. While lists developed from expert opinion can be useful, evidence suggests that empirical validation is an important step in demonstrating the usefulness of quality measures [20]. Ongoing work as part of the Every Newborn Action Plan is seeking to validate the signal functions of small and sick newborn [21]. Furthermore, the SPA surveys were not designed to measure routine and EmNC functions proposed by the framework of Gabrysch and colleagues. As a result, we used proxy-measures derived from the presence of tracer items to assess the facilities’ abilities to perform routine functions. These routine functions, as shown in Table 4, were said to be conducted routinely in a facility if the facility representative interviewed endorsed it as routine. While it is helpful that the survey included information on what interventions are said to be conducted in an uncomplicated delivery in any given facility, confirming the routine nature of these interventions (i.e., through observation) would have been helpful, as there are well-documented differences between self-reported practices and observed practices [20]. While including observation of necessary tracer items for each signal function is a strength, cross-sectional observations such as the SPA may be criticized for providing only a point-prevalence in availability of these items, which is arguably problematic when attempting to assess a busy and dynamic facility environment [22].

Furthermore, simply because a tracer item is present does not mean it will be used in the correct manner or in the correct patient at the correct time, and performance of any given signal function may be appropriate or inappropriate. It is inadequate to base care quality assessments on provider knowledge or facility capability alone, as avoidable deaths may occur if the resources are not used or used incorrectly [23–24]. Thus, a weakness in this entire signal function approach is that it cannot measure the appropriateness of the care provided, only the binary measure of whether interventions were reportedly capable of being provided or not. The signal function approach measures what is necessary to provide care, but in an of itself, is not sufficient to ensure all women and babies get care. To assess the latter, one approach has been to ask if care was provided in a given period of time, for example in the last six months, but even this is not actually good enough to assess if care was provided each time it was needed. Moreover, such an approach is even less adequate to measuring care that should be given to all women, such as infection prevention or active management in third stage of labor. Another approach has been to ask about the frequency with which care was provided, along a Likert scale (i.e. always, most times, sometimes, rarely, never) [25].

Similarly, there are limitations to the conventional “performed in previous three months” measurement, as it favors facilities with higher delivery caseloads since they are more likely to see individual maternal and newborn complications. Smaller facilities that may indeed be prepared to manage such complications but do not have the delivery caseload to see individual complications within a given time period would therefore not meet criteria for capability to provide that signal function. One could argue, however, that facilities truly need to see a complication more often in order to maintain the skills to manage said complication [5]. Furthermore, while it makes sense that facilities with higher delivery traffic would meet criteria for the emergency functions in the past three months, patients in such facilities may also face high patient-provider ratios that preclude consistent delivery of routine and emergency interventions. Finally, measurements of the more complex CEmOC functions of blood transfusion and cesarean section were not fully developed in this study. While these functions are life-saving, we chose to focus on the less complex BEmOC functions, as more facilities in low resource settings are, by definition, able to provide BEmOC functions than CEmOC functions. Furthermore, one could argue that both CEmOC functions require personnel and infrastructure of higher cadre than the BEmOC functions.

The delivery caseload weight methodology proposed has some potential flaws, particularly if the data for delivery caseload are unreliable. Also, there is a lack of metrics for measuring other relevant factors such as facility crowding, time spent at the facility and whether the quantity of specific tracer items is sufficient for the caseload. It is also important to remember that while it is logical to assume that women and babies in a facility incapable of providing a signal function will not receive it, it does not follow that all women and babies in a facility capable of providing a signal function will receive it if and when they need it. Thus, validation of the relationship between this structural component of the Donabedian framework and the corresponding process aspects is essential for establishing the usefulness of this method [10]. Lastly, it should be noted that our new methodology does not include measuring dimensions of facility staffing, not because proper staffing is not an issue, but rather because staffing numbers are not crucial in shifting the denominator of our metrics from facilities to individual deliveries. As part of future research, analyses could be done assessing proper staffing instead of or in addition to delivery signal functions (e.g. “x% of deliveries took place in a facility with less that the number of recommended midwives”).

Lastly, we must remember that the percentage of deliveries that take place outside of health facilities can vary greatly by geographical location. In many countries, looking at the quality of childbirth care in health facilities is merely the tip of the iceberg because so many deliveries take place outside of facilities. We reiterate that our conclusions only apply to the 43% of births in Kenya that took place in a health facility [13].

### Emergency childbirth care capabilities

When we required tracer items to be available in addition to the BEmOC criteria of having performed a function in the previous three months, the overall proportion of facilities equipped to perform each individual function decreased. However, the decrease was not uniform across all facility levels. Capabilities of hospitals decreased relatively little, whereas health centers, clinics and dispensaries were especially hard-hit by the added criteria. This is consistent with the literature which says that these lower-level facilities have the largest gap between service requirements and service provision [26–28]. Moreover, it is questionable as to whether a facility with a small caseload of deliveries should be expected to have even encountered certain complications with in a three-month period, if the prevalence of the complication is low.

The picture for EmNC was worse than that for EmOC: only 3% of facilities could provide BEmNC or CEmNC (Table 5). CEmNC capability is likely even lower than our study suggests because placing an IV in a newborn is quite a difficult task and it can be assumed that in many facilities that had the necessary tracer items to provide IV fluids to a newborn, many would not be able to carry out the task. While antibiotics for preterm premature rupture of membranes and/or sepsis were available in roughly half of facilities, only 35% of facilities were capable of providing neonatal resuscitation and even fewer (21%) were capable of providing corticosteroids during preterm labor (Table 3). It must be noted that, although antibiotics were the most widely available EmNC function, only 30% of facilities had adequate infection prevention measures during childbirth (Table 4). Thus, the continuum of infection prevention for mother and newborn was inconsistent. Contextualized within the most recent newborn mortality data released at the time the SPA data were collected, in which the leading causes of neonatal mortality were infection (29%), prematurity (29%) and asphyxia (23%) [29], the wide gaps in facility capability to perform functions that directly prevent, treat or decrease the burden of these problems is especially concerning.

### Routine childbirth care capabilities

Of the dimensions of quality care, facilities overall performed the best in general requirements and routine maternal functions (both 13%) (Table 4). While the vast majority of facilities had communication tools (92%), electricity (83%) and latrines (99%), an alarmingly low 15% of facilities had a high-quality referral system (Table 4). While the criteria for referral system may seem strict, efficient referral systems are essential because most facilities were not equipped to perform all BEmOC-1 and BEmNC functions. It may be, however, that requiring lower level facilities to have vehicles is not necessary, if emergency medical service vehicles are located at larger facilities that go to lower level facilities or if they are located at a mid-point and directed by call centers.

Routine maternal care overall had reasonable levels of performance for specific functions. The three functions of active management in third stage of labor were performed on a routine basis in most facilities, likely preventing many life-threatening cases of postpartum hemorrhage. Most concerning were low levels of effective infection prevention measures. While 92–100% of hospitals had piped water, only 17–52% of non-hospitals had a clean water source (Table 4). Only 30% of facilities had proper infection prevention measures. This is concerning, as 10% of maternal deaths [30] and 36% of neonatal deaths [31] are due to infection. Among routine newborn functions, facilities were least equipped to provide thermal protection: drying baby after birth (30%) and wrapping the baby in a blanket (27%). It is possible that blankets are brought by mothers, and so we may have been unnecessarily strict with this criterion.

### Implications for using delivery caseload weights

When the delivery caseload weights were applied, the coverage of facility preparedness appeared to improve. This is because more deliveries take place in higher-level facilities, which tend to be more likely to meet criteria for delivery preparedness. Evidence from the United States has shown a positive correlation between delivery caseload and improved maternal and neonatal outcomes with increased complication rates at facilities with very low caseloads as well as those with exceedingly high caseloads [32]. However, sufficient evidence is lacking for this relationship in low income countries. Applying the caseload weights in our study did improve perceived delivery preparedness, so we argue that using facility capabilities alone in an attempt to operationalize the care capability at the place of delivery at a national level is insufficient, and potentially underestimated the quality of care received and the extent to which births are in a context that can manage routine care and complications. Applying this methodological lens is a crucial step forward in utilizing metrics for tracking maternal and newborn health preparedness across regional, national and cross-national boundaries. Utilizing these metrics can enable cross-national comparisons that produce a more standardized method of investigating the phenomenon of delivery preparedness. We found this to be particularly important in the category of “other hospital” (those hospitals which did not fit the categorization of national referral, provincial, district or sub-district, all of which are predominantly government-managed) which had enormous variability in the content of care as well as the number of deliveries taking place in each facility, ranging from eight to nearly 18,000 (Fig 3). While traditional survey weighting techniques may be less problematic when facilities of a specific type have similar performance and caseload (e.g., if all district hospitals performed approximately 5000 deliveries per year) they become less useful when the facility categorization, such as facility type, does not differentiate between high caseload and low caseload facilities. The delivery caseload weights methodology answers part of a wider call for the use of comprehensive facility assessments and facility utilization data to move from merely monitoring coverage to monitoring “effective coverage” of essential interventions that are more likely to align accurately with health outcome measures, such as reduction in morbidity and mortality among mothers and newborns [1, 8, 33].

### Further research

Facility assessment surveys, such as the SPA, are greatly under-utilized. Considering the richness of the data used in this study, we would advocate for broader utilization of facility assessment surveys, specifically to characterize provision of routine and emergency obstetric and newborn care. In this study, we did not attempt to capture the nuances of provision of more complicated functions, such as caesarean section or prevention of mother to child transmission of HIV. These services are investigated extensively in the SPA surveys and we would suggest more effort be applied to characterizing facility capabilities to perform these complex yet life-saving interventions. Furthermore, types and numbers of facility staff are detailed in SPA surveys and it could be useful to integrate delivery caseload with patient-provider ratios to elucidate delivery caseloads not just per facility, but per provider as well.

In order to add analytical dimensions of facility utilization and crowding, we advocate for the inclusion of delivery caseload, number of beds and median time in facility to be included in all facility assessments. We would further suggest that the method of delivery caseload weights be utilized in large facility assessments and further developed to include measures of uncertainty. Validation of this methodology is necessary, as quality assessment is predicated on the existence of a relationship between measured structures and processes (as well as structures and outcomes) [10]. It must be established that: 1) capability to perform routine or emergency care functions is associated with correct performance of such functions in the appropriate patient at the right time (structure associated with process); 2) improved facility preparedness using the delivery caseload weights methodology is more closely associated with decreased maternal and neonatal mortality than using facility weights alone (structure associated with outcomes), as these are true measures of impact.

## Supporting information

S1 Table A comparison of the traditional weighting method versus the delivery caseload method for assessing facility preparedness. (DOCX)
