# Supplementary material for: Measuring facility capability to provide routine and emergency childbirth care to mothers and newborns: An appeal to adjust for delivery caseload of facilities
Source: PLoS One. 2017 Oct 19;12(10):e0186515. doi: 10.1371/journal.pone.0186515 (PMC5648263; doi:10.1371/journal.pone.0186515)
Supplement: S1 Table — (DOCX) [file pone.0186515.s001.docx]

**S1 Table: A comparison of the traditional weighting method versus the delivery caseload method for assessing facility preparedness**

| **Method** | **Formula** | **Example:** Health Centre in Rift Valley province with 904 deliveries in past 12 months |
| --- | --- | --- |
| Traditional weighting method | Weighted proportion of total facilities in the dataset represented by a specific facility (due to over- or under-sampling of each facility type within a given region):  $=\frac{facility weight}{\sum values of facilitiy weights}$ | Traditional survey weight for a health centre in Rift Valley Province is 1.652  The weighted proportion of all facilities in the dataset represented by this facility is:  = $\frac{1.652}{207.33 total weighted facilities\#}$  = 0.007  Each health centre in Rift Valley province that was included in the survey has the same facility weight (1.652) even though the number of delivery clients in the past 12 months ranged from 16 to 904. |
| New delivery caseload weights method | New weight variable for a specific facility:  $=\frac{\left( facility weight \right) \times\left( no. of delivieres in facility in past 12 months \right)}{\sum total no. of delivieres}$ | Caseload weight:  =$\frac{\left( 1.652 \right)*\left( 904 deliveries \right)}{61459.64 total deliveries in dataset}$  = 0.243  This is the proportion of weighted total deliveries in the dataset that took place in this facility and is thus the new weight variable |
|  | | |

#The sum of all facility weights included in the assessments does not equal 695, as 403 of the 695 facilities included offered delivery care and hospitals and other higher level facilities were over-sampled in this sampling frame, thus having lower facility weight values (many of which are <1.0). The sum of all 403 facility weight values was 207.33.
